# Characterizing Sleep Spindles in Sheep

**DOI:** 10.1523/ENEURO.0410-19.2020

**Published:** 2020-03-06

**Authors:** Will T. Schneider, Szilvia Vas, Alister U. Nicol, A. Jennifer Morton

**Affiliations:** Department of Physiology, Development and Neuroscience, University of Cambridge, Cambridge CB2 3DY, United Kingdom

**Keywords:** automated detection, awake EEG, learning, slow waves, spectral analysis, wake spindles

## Abstract

Sleep spindles are distinctive transient patterns of brain activity that typically occur during non-rapid eye movement (NREM) sleep in humans and other mammals. Thought to be important for the consolidation of learning, they may also be useful for indicating the progression of aging and neurodegenerative diseases. The aim of this study was to characterize sleep spindles in sheep (*Ovis aries*). We recorded electroencephalographs wirelessly from six sheep over a continuous period containing 2 nights and a day. We detected and characterized spindles using an automated algorithm. We found that sheep sleep spindles fell within the classical range seen in humans (10–16 Hz), but we did not see a further separation into fast and slow bands. Spindles were detected predominantly during NREM sleep. Spindle characteristics (frequency, duration, density, topography) varied between individuals, but were similar within individuals between nights. Spindles that occurred during NREM sleep in daytime were indistinguishable from those found during NREM sleep at night. Surprisingly, we also detected numerous spindle-like events during unequivocal periods of wake during the day. These events were mainly local (detected at single sites), and their characteristics differed from spindles detected during sleep. These “wake spindles” are likely to be events that are commonly categorized as “spontaneous alpha activity” during wake. We speculate that wake and sleep spindles are generated via different mechanisms, and that wake spindles play a role in cognitive processes that occur during the daytime.

## Significance Statement

Sleep spindles provide an indication of brain health and function. In this study, we characterize sleep spindles in sheep (*Ovis aries*) for the first time. We found that sleep spindles in sheep are similar to those found in humans in many respects (e.g., density, duration, and frequency) and occurred mainly during non-rapid eye movement sleep. Interestingly however, we also saw spindles during wake in the day. Spindles detected during wake were characteristically distinct from those occurring during sleep. We suggest that wake and sleep spindles are generated via different mechanisms and may have different functional roles. Wake spindles may be a component of cognitive processes that occur during the daytime, such as memory retrieval and attention.

## Introduction

Sleep spindles are transient and distinctive patterns of brain activity that typically occur during non-rapid eye movement (NREM) sleep in humans. They have been documented in several other animal species, including mice (Kim et al., 2015), rats (Eschenko et al., 2006), dogs (Iotchev et al., 2017), cats (Contreras and Steriade, 1996), monkeys (Takeuchi et al., 2016), and ferrets (McCormick and Bal, 1997). Sleep spindles are considered to play a key role in memory consolidation and have been studied in relation to both learning ability and cognitive impairment (Clemens et al., 2005; Eschenko et al., 2006; Iotchev et al., 2017). Interplay among multiple circuits (the thalamus, cortex, and hippocampus) is known to result in spindle generation (Lüthi, 2014). These same circuits are active during learning in wake (W; Vukadinovic, 2011). Therefore, it is thought that the functional status of these brain networks can be inferred by the characteristics of spindles observed in EEG recordings (Lüthi, 2014; Ciric et al., 2017).

The quantitative definition of a sleep spindle is not universally agreed upon, although there is a consensus of opinion that sleep spindles occur in the 10–16 Hz frequency range (Gibbs and Gibbs, 1941; Andrillon et al., 2011). Spindles are characterized by a waxing and waning shape and are generally considered to last between 0.3 and 3 s (Gibbs and Gibbs, 1941). In humans, the classical 10–16 Hz spindle band is often subdivided into fast (>13 Hz) and slow (<13 Hz) spindles. This distinction is made because fast and slow spindles are considered to be generated via different mechanisms (Mölle et al., 2011; Ayoub et al., 2013), resulting in different temporal relationships to slow waves (SWs; Mölle et al., 2011). In sleep, the most prominent EEG feature is the presence of SWs, reflecting an oscillation between the activated (upstate) and inactivated (downstate) states of cortical neurons (Contreras and Steriade, 1995). In humans, fast spindles occurring on the SW upstate are thought to have a closer association with memory and learning than slow spindles that occur on the SW downstate (Mölle et al., 2011). Fast spindles are found predominantly in the centroparietal cortex, while slow spindles are more dominant at frontal regions (Andrillon et al., 2011). Among animal models, however, the distinction between fast and slow spindles is less clear. For example, in the macaque (*Macaca fuscata*) the fastest spindles are found in the frontal and central regions, while slow spindles are found predominantly at the back of the cortex (Takeuchi et al., 2016). This is the opposite to that found in humans. The apparent function of fast and slow spindles also varies. In dogs, only slow spindles (<13 Hz) predict learning ability (Iotchev et al., 2017), while in rats only fast spindles (>12 Hz) correlate with learning (Eschenko et al., 2006).

Here we used sheep (*Ovis aries*) as a large animal model to study sleep spindles. Sheep have a number of advantages over other species for studying the neurobiology of sleep. They have human-like brain anatomy with elaborately convoluted cortices. They are diurnal, and their sleep structure is more similar to humans than that of rodents (Toth and Bhargava, 2013). Their husbandry and welfare is easily managed, particularly when compared with nonhuman primates (Morton and Howland, 2013). Techniques for EEG monitoring in sheep are already well established (Perentos et al., 2016), and telemetric data collection allows continuous longitudinal recording across night and day. We used automated detection methods to identify spindle characteristics in sheep from recordings made continuously over a 34 h period. We analyzed our data according to sleep state and provide a detailed characterization of sleep spindles in sheep. We show that many of the measures used to characterize human spindles can be used in sheep, and that these measures provide consistent markers of an individual’s brain state between different nights. Because we recorded continuously, we were also able to make direct comparisons of spindles that occur during day and night, and between wake and sleep occurring in both of these periods. We recorded local spindles during wake and provide evidence to show that these are characteristically divergent from the spindles that typically arise during NREM sleep.

## Materials and Methods

### Sheep

Six female merino sheep were used for these recordings. All procedures were conducted at the Preclinical Imaging and Research Laboratories of the South Australian Health and Medical Research Institute (SAHMRI) and followed the requirements of the SAHMRI Animal Ethics Committee including the Australian Code for the Care and Use of Animals for Scientific Purposes (eighth edition). All sheep were genetically normal but were part of a flock that included transgenic animals. Accordingly, all handling of these sheep conformed to physical containment conditions as approved by the Institutional Biosafety Committee and the Office of the Gene Technology Regulator (OGTR, Australia). At the time of surgery, sheep were ∼6 years of age, and their mean weight was 85 ± 3 kg. After surgery, they were housed together in a covered outdoor area with natural lighting, in individual pens separated by smooth transparent Perspex partitions.

### Surgery

**Figure 1.**
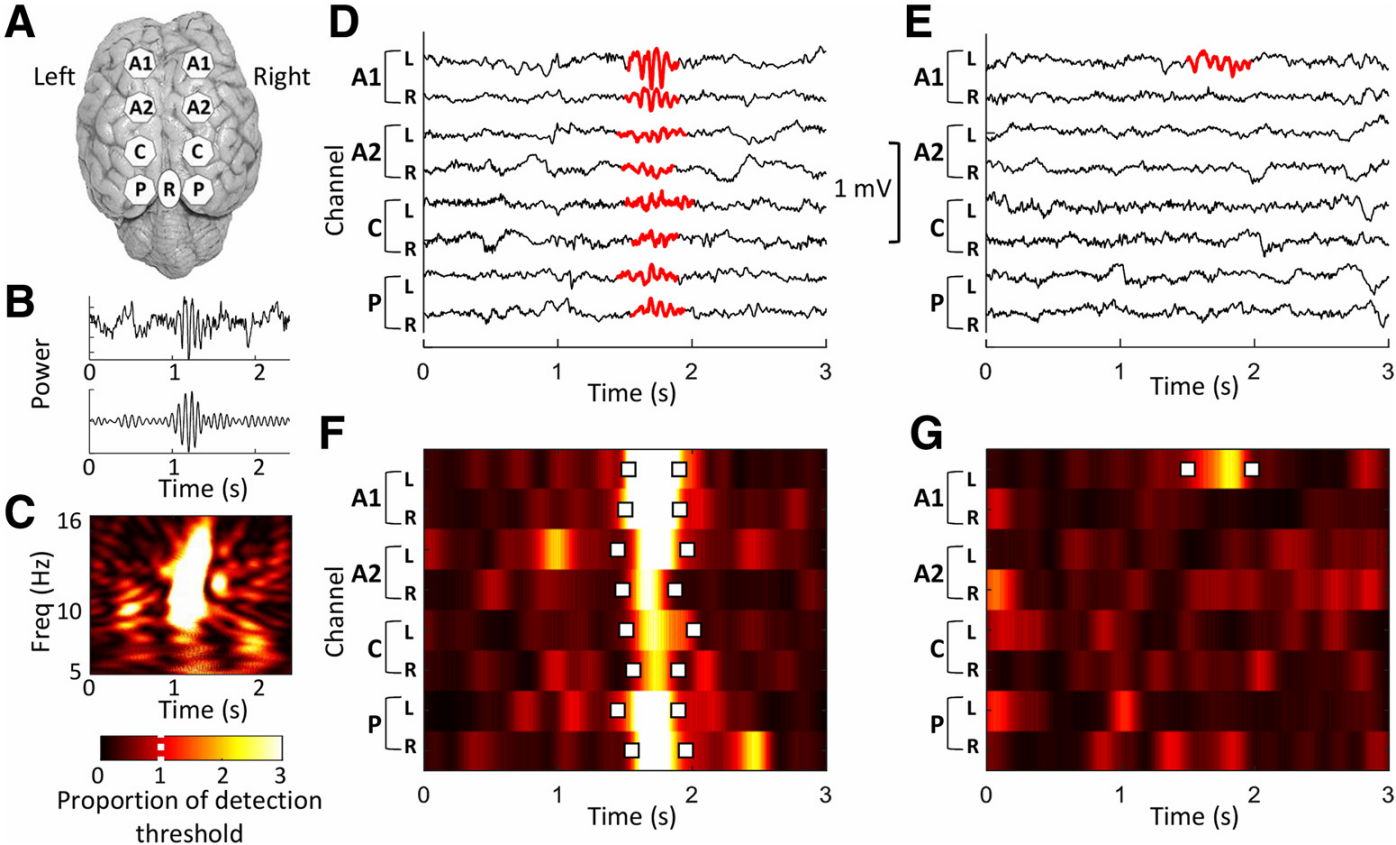
Overview of spindle detection in sheep. ***A***, Eight recording channels and a reference electrode were surgically implanted onto the surface of the sheep brain. ***B***, Sleep spindles were observed in both raw waveforms (top) and when filtered between 10 and 16 Hz (bottom). ***C***, A spectrogram of the sleep spindle in ***B***. ***D***, ***E***, Raw traces for each of the eight recording channels show an example of a global sleep spindle event (highlighted in red) occurring simultaneously in multiple channels (***D***), and a local sleep spindle occurring in a single channel (***E***). The color scale shows the proportion of the detection threshold. This is scaled to the power in each channel so that a value of “1” represents the power threshold that needs to be exceeded in order for a spindle to be detected. This figure is extended in Extended Data Figures 1-1 and 1-2. R, Reference.

The surgery for implanting electrodes was conducted as described by Perentos et al. (2016, 2017). Briefly, subdural electrodes (3 mm diameter × 1 mm deep Ag/AgCl discs; NDimension (Science and Engineering) were implanted at sites across the cortex (Fig. 1*A*). One of these, positioned at the midline over the transverse sulcus, served as a reference electrode. Eight recording electrodes were positioned bilaterally at locations corresponding approximately to the postcruciate gyrus [anterior 1 (A1)], the ansatus sulcus [anterior 2 (A2)], the front third of the ectolateral sulcus [central (C)], and the lateral sulcus near the anterior part of the entolateral sulcus [posterior (P)]. Stainless steel coils were implanted in the dorsal splenius muscles to record neck electromyography (EMG). Electrodes also were positioned at the inner and outer canthi of each eye to record the electrooculogram (EOG). A subdural electrode for online referencing was positioned 10 mm posterior to the bregma at the midline. Wires from all electrodes were terminated at a nano-miniature multipin male strip connector (NPD-18-WD-18.0-C-GS, Omnetics Connector Corporation). When all electrodes were implanted, the connector and wires were enclosed in a 3D-printed polyamide (nylon) chamber (G.E. Baker) fitted to the head of the sheep. During recordings, a top stage was fitted to the chamber. This stage held a transmitter amplifier (W2100-HS16, Multichannel Systems), mated to the Omnetics Connector Corporation connector, and a battery capable of supporting recording for ∼24 h. Signals were transmitted wirelessly. Recordings were conducted 1–4 months post-surgery.

10.1523/ENEURO.0410-19.2020.f1-1 Figure 1-1 Examples of intersheep differences in spindle characteristics. Whisker plots show spindle characteristics during NREM for each sheep (*N* = 6). ***A–D***, Spindle density (***A***) frequency (***B***), spindle length (***C***), and spindle power (***D***). All data are taken from night 2. Spindle characteristics vary widely between individuals, and this is not due to a single outlier. Download Figure 1-1, TIF file.

**Figure 2.**
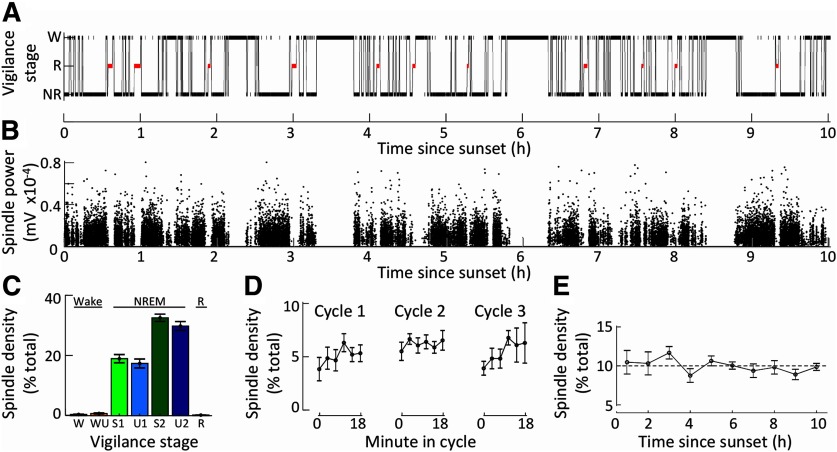
Spindle density correlates primarily with non-rapid eye movement sleep. ***A***, A hypnogram for a single sheep is shown between sunset (*t* = 0) and sunrise (*t* = 10). Data are shown as the mean ± SEM. ***B***, Power of spindle detections from the same EEG recording. A black dot marks each spindle detection. Detections from all channels are included. ***C***, The proportional density of spindles detected (*N* = 6 sheep) in each vigilance stage. ***D***, Proportional densities (*N* = 5 sheep) of spindles for the first three sleep cycles lasting 18 min. ***E***, Proportional densities (*N* = 6 sheep) of spindles throughout a single night (normalized for the amount of NREM sleep in each hour period). W, wake; WU, wake with rumination; R, REM sleep (highlighted in red); NR, NREM sleep. NREM sleep is split into four subgroups (light green, S1; light blue, U1; dark green, S2; dark blue, U2).

10.1523/ENEURO.0410-19.2020.f1-2 Figure 1-2 General spindle characteristics in sheep. Download Figure 1-2, DOCX file.

### Recording

Recordings were made using a wireless telemetry system (Advanced W2100-System, Multichannel Systems) at a sampling frequency of 1 kHz. EEG data were recorded from eight cortical electrodes that were designated left and right A1 (A1-L, A1-R) and A2 (A2-L, A2-R), left and right C (C-L, C-R), and left and right P (P-L, P-R) channels (Fig. 1*A*). EOG and EMG data were also recorded. Battery changes to the on-sheep telemetry equipment were needed for continuous recording. We chose to do this during the middle of the day at the same time as facility animal husbandry activities were performed. As a result, this period in the middle of each day was omitted from the analysis. Thus, for the day, 9 h recordings were used that comprised the first 4 h after sunrise and the last 5 h before sunset. The night recordings were uninterrupted.

### Preprocessing

Data were collected in serial 1 h epochs. These were then downsampled to 250 Hz. Once downsampled, they were compiled offline into three files (2 nights and 1 d). The EEG data were rereferenced offline using a common average reference for all EEG channels that was subtracted from each channel. Recordings were then imported into MATLAB for spindle detection and characterization.

### Code accessibility

The code/software described in the article is freely available online at [https://uk.mathworks.com/matlabcentral/fileexchange/73390-spindle-characterisation-characterising-sleep-sp-in-sheep]. The code is available as Extended Data Code Files.

10.1523/ENEURO.0410-19.2020.ed1 Extended Data 1 - Code Files The spindle detection code is included here. The file “basicSpindleWorkflow” gives an example of how the code can be run. The code is also available online at the following URL: [https://uk.mathworks.com/matlabcentral/fileexchange/73390-spindle-characterisation-characterising-sleep-sp-in-sheep]. Download Extended Data 1, ZIP file.

### Spindle detection

Automated spindle detection was performed using custom MATLAB scripts. The detection algorithm was run in MATLAB 2018b, on a 64 bit Windows 10 desktop computer. This was originally based on the SWA-toolbox spindle-detection functionality (Mensen et al., 2016). The adaptations we made to this method include numerous alterations to how spindles were detected, characterized, and processed, as well as data management (see Extended Data Code Files). In brief, recordings were imported into MATLAB from a text file. In the first processing step, following the SWA-toolbox methodology, a wavelet transform obtained frequency powers between 5 and 16 Hz (Friess et al., 2015). Next, possible spindles were highlighted if the mean power within this range exceeded a threshold (calculated separately for each channel and each sheep: 180% of the mean 5–16 Hz power in the night 2 recording). Although night 1 and night 2 recordings were very similar, the night 2 recording was used as the “baseline” to avoid a possible first-night effect (Toussaint et al., 1995). We made changes to the SWA-toolbox method by altering the way in which the start and end points of spindles were determined to avoid instances where single spindles were incorrectly detected as separate events or where multiple spindles were detected as single spindles. In our method, the start and end of each spindle was defined by locating the point at which the mean spindle-range power dropped to <80% of the prominence of its peak, or <120% of the channel mean power. Next, a discrete fast Fourier transform (FFT) using a Hanning window and 1024 frequency bins for each spindle was used to determine power spectral density and to find the peak frequency and peak frequency power. Detected spindles lasting <0.3 or >3 s were discarded. Spindles with a sufficiently high power within the detection range but with a peak frequency outside of this range were also discarded. Noise spikes were removed if they contained sudden spikes of power >10 times the mean variation in voltage in the spindle. Finally, in order to be included, a filtered spindle needed to contain at least three positive peaks. We allowed our algorithm to detect spindles between 5 and 16 Hz because spindles in other animal models have been found at lower frequencies than those in humans (Contreras and Steriade, 1996; Iotchev et al., 2017). However, we found no evidence of a clear separation in spindle frequency bands (data not shown); therefore, in this article we have focused on spindles falling into the classical range of 10–16 Hz.

#### Sleep stages

Sleep scoring was performed with semiautomatic analysis using SleepSign software (Kissei Comtec) for the night 2 and day periods (Fig. 2*A*). Seven vigilance stages were separated based on previously published criteria (Perentos et al., 2016). W was defined by low-voltage high-frequency EEG accompanied by increased EMG activity and intensive but irregular ocular movements. W with concurrent rumination (WU) was additionally defined by EOG and EMG signals containing regular mastication (Perentos et al., 2016). NREM sleep was characterized by high-voltage SWs, decreased muscle tone compared with W, and occasional slow rolling eye movements. In NREM sleep with concurrent rumination (U), EMG and EOG channels contain signals consistent with mastication. Based on the delta power, NREM sleep was further separated into “light NREM sleep” (S1) and “deep” NREM sleep (S2). We also differentiated light NREM sleep accompanied by U (U1) and deep NREM sleep accompanied by U (U2). The threshold between S1/S2 and U1/U2 was defined based on 50% of the maximum delta power. During REM sleep, low-voltage high-frequency EEG activity was accompanied by rapid ocular movements appearing in bursts. Muscle tone in this stage is reduced compared with NREM sleep but is sometimes interrupted by transient muscle activity. Quantitative EEG analysis shows no difference between sleep states with or without rumination (data not shown). The scored data were used to investigate differences in spindle characteristics between vigilance stages, excluding any epochs that had been flagged as containing artifacts.

#### Sleep cycles

Sleep cycles were automatically identified using the results from the semiautomatic sleep stage scoring. Sleep cycles in sheep are shorter than those in humans (Perentos et al., 2016). We defined sleep cycles as starting after a minimum of 30 s of wake, followed by a period of NREM sleep lasting at least 2 min that terminated either with wake or at least 1 min of REM sleep. Spindle density within sleep cycles was compared by examining the first three sleep cycles for each sheep that lasted at least 18 min. This length of sleep cycle was chosen empirically as it was long enough to quantify spindle density changes, but not so long as to be a rare occurrence. Each sleep cycle was split into 3 min periods. Spindle density during each 3 min period was quantified. Periods of 3 min allowed enough time for reliable and stable counts of spindles. Spindle densities were then normalized as proportions of the total number of spindles detected for each sheep.

#### Spindle density throughout night

Spindle density was calculated for each hour period of night 2. This was then normalized (for every channel, in each sheep) according to the amount of time spent in NREM sleep in each hour period.

#### Spindle–slow wave relationship

To detect SW activity, EEG was filtered between 0.5 and 2 Hz (Bersagliere and Achermann, 2010). To find the SW peak-to-peak amplitude, a 3 s window either side of every spindle center was created. The positive and negative peaks on the SW located closest to the spindle centers were found. The difference between these two peaks gave the SW peak-to-peak amplitude for each spindle.

### Simultaneous spindles

Spindles frequently occurred in multiple channels. For every spindle, any spindle in another channel that overlapped with it in time was classified as occurring “simultaneously”. The number of spindles occurring simultaneously for each spindle was recorded, as were the identities of the channels in which each spindle occurred. Spindles can therefore be differentiated into those that occurred “locally” (i.e., in just one channel at a time) or simultaneously (i.e., in more than one channel). Simultaneous spindles were then further separated into the following groups: those occurring in two channels simultaneously (to investigate links between any two particular channels) or those occurring in four or more channels simultaneously (to look at widespread global spindle events). We have developed a method for visualizing these data in two different ways. First, for every sheep, the number of simultaneous spindles shared between channels was calculated as a proportion of the total number of simultaneous spindles. Second, the number of simultaneous spindles was quantified as a proportion of the total number of simultaneous spindles in each channel separately.

### Night/day and sleep/wake spindle differences

Spindles were classified by time of occurrence into night or day spindles, and by vigilance stage. This produced the following four vigilance groups: night sleep, day sleep, night wake, and day wake. Characteristics of spindles were compared in each group. These were spindle density, mean frequency, mean duration, and mean spindle–SW phase angle.

### State space analysis for spectral properties of day wake spindles

State space analysis is a technique used to investigate boundaries and transitions between states of consciousness (Diniz Behn et al., 2010). We used it to determine whether wake epochs that contained spindles were any “sleepier” than general wake epochs. To do this, we calculated the ratios of power detected in different frequency bands for every epoch. We used two power band ranges to calculate the state space ratio (SSR). For SSR1, we used 6.5–9/0.5–9 Hz, and for SSR2 we used 0.5–20/0.5–100 Hz. These ratios were based on previous studies conducted on mice (Diniz Behn et al., 2010). Our ranges differ from those used by that group because we increased the upper bound of SSR2 to 100 Hz, to use the high frequencies of our recordings. We found no reason to change these ratios further as they provided clear separation between the NREM sleep and wake states. To form an “all epoch” state map, all epochs (10 s) scored as wake or NREM sleep were collated for an entire night (3731 potential epochs per sheep). FFTs were performed, as before, on all raw EEG channels within each epoch. The mean power was calculated for all channels, and then the state space ratios for each epoch were determined. To form the spindle–occurrence state map, 10 s epochs centered on every spindle occurrence were collated for all local spindles occurring in both wake and NREM sleep. The ratios were then calculated in two different ways. The first was the same as for the all epoch state space map; the mean of the FFT output was calculated from all channels. In the second, instead of taking the mean of all channels only the FFT output from the channel that contained the detected spindle was used. To create density maps, the two-dimensional ratio space was gridded and the number of epochs with ratios falling in the state space of each grid unit was summed. The grid unit size was 0.025. For each sheep, the values within each grid unit were divided by the total number of epochs, giving a proportional density per grid unit (totaling 1). The maps for all six sheep were then summed together, such that the sum of all the units in this new grid space equaled 6. The grid space size was 41 × 41, giving a total of 1681 grid units. For purposes of visualization, thresholds were used to indicate contours around the highest density areas. If the density spread was even throughout the grid, each grid would contain a value of 6/1681. The lower and upper thresholds were set as 2.5 × (6/1681) and 5 × (6/1681), respectively. These values were chosen because they provided an easily interpretable indication of the extent of the wake and NREM sleep states.

### Statistical analysis

All statistical analyses were performed in RStudio (version 1.1.463). Unless otherwise stated, data are shown as the mean ± SEM. One-way repeated-measures ANOVAs were used to test differences in spindle characteristics between sheep and channels (repeated factor). Levene’s test was used to check the homogeneity of variance. Data were logged where necessary to achieve normal distributions. To investigate spindle density during sleep cycles or throughout the night, density values were normalized in each channel by calculating the densities as proportions of the total density during the three cycles, or the entire night, respectively. Two-way ANOVAs were used to test for differences in proportions of spindle density between sleep cycles or 3 min windows within each sleep cycle. Five of the six sheep had three sleep cycles lasting >18 min; one sheep had only two sleep cycles >18 min. Therefore, data from this sheep were not included in the sleep cycle density analysis. A one-way ANOVA was used to test differences in proportional spindle density between hours in the night. Wilcoxon signed-rank tests were used to make pairwise comparisons between vigilance groups to test spindle density differences for local and simultaneous spindles because the variance between groups in these data were not homogeneous. Generalized linear models using γ distributions were used to test the correlation in spindle density between vigilance groups. Normal distributions allowed linear models to be used to test correlations in mean spindle frequency and duration between vigilance groups. Sleep scoring information was not needed for testing the difference in SW peak-to-peak amplitude between local and simultaneous spindles; therefore, this analysis could be performed for all recording periods (as a repeated factor). A *p* value or α value <0.05 was accepted as significant throughout.

## Results

### Spindle detections

Our algorithm detected sleep spindles in the sheep EEG within the 10–16 Hz classical range (Fig. 1*B*,*C*). We found spindles as both global events occurring in all channels (Fig. 1*D*,*F*) and local events occurring in only a single channel (Fig. 1*E*,*G*). Mean spindle density for all sheep (*N* = 6) during NREM sleep ranged from 5.9 ± 1.3 spindles per minute (channel A1-R) to 3.5 ± 0.3 spindles per minute (channel P-L; Extended Data Fig. 1-2). There was a nonsignificant trend for spindle density per minute of NREM to vary between channels (*F*_(7,35)_ = 2.0, *p* = 0.076; Extended Data Fig. 1-2). Spindles were present in the entire 10–16 Hz frequency range. Spindle frequency differed between channels (*F*_(7,35)_ = 2.8, *p* = 0.019), although this was driven solely by lower-frequency spindles in the C-R channel (Extended Data Fig. 1-2). Neither spindle duration nor power differed significantly between channels (Extended Data Fig. 1-2).

Individual variance between sheep in spindle characteristics was high. We found that spindles differed significantly between sheep in density (*F*_(5,35)_ = 9.7, *p* < 0.0001), frequency (*F*_(5,35)_ = 13.6, *p* < 0.0001), duration (*F*_(5,35)_ = 2.9, *p* = 0.028), and power (*F*_(5,35)_ = 5.1, *p* = 0.0013). This was not due to a single outlier sheep (Extended Data Fig. 1-1).

### Macrostructure

At night, spindle density occurred almost exclusively during NREM sleep episodes (Fig. 2*A–C*). The presence of rumination during a sleep stage did not alter any spindle characteristics. Spindle density did not change during or between the first three sleep cycles (Fig. 2*D*) and remained stable throughout the entire night (Fig. 2*E*).

### Simultaneous spindles

**Figure 3.**
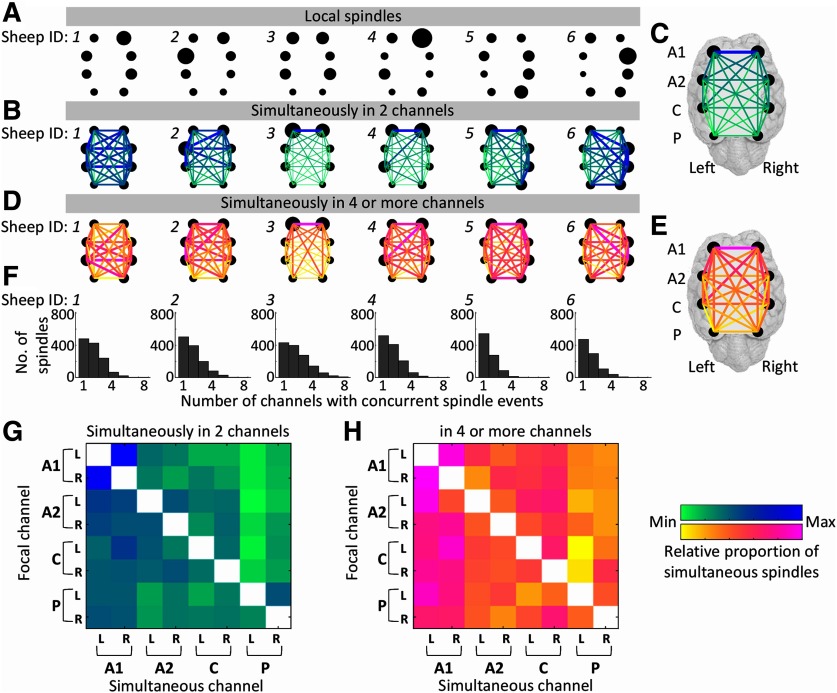
Local and simultaneous spindle topography. ***A***, Spindles that occurred locally (only in one channel) are shown as the proportion of the total number of local spindles that occurred, for each sheep. The size of each black dot represents the number of local spindles occurring in a particular channel. ***B***, The data represent the occasions on which spindles occurred simultaneously in two channels. The size of the black dot is again representative of the number of spindles occurring at each electrode for each sheep. The pairing of spindles is shown by the color and size of the lines linking two dots. Thicker lines and darker colors mean the likelihood of spindles firing simultaneously in those two channels is higher. ***C***, The mean of spindle pairings for all sheep (*N* = 6). ***D***, Data show the distribution of spindles when four or more spindles occur simultaneously. Again, the proportion of spindles occurring in a particular channel is shown by the size of the back dot, and the likelihood of simultaneous occurrence of spindles is shown by pseudocolored lines linking the dots. ***E***, Mean data for spindles occurring simultaneously in four or more channels are shown (all sheep; *N* = 6). ***F***, Histograms show how many channels were involved in each spindle event, for each sheep. ***G***, ***H***, For pairs of spindles (***G***) and for spindles occurring in four or more channels (***H***), the relationship between the channels in which simultaneous spindles occurs is shown as heat maps. For ***G***, where data from two simultaneous spindles are shown, the rows represent the total number of spindles occurring in a particular channel, and the columns show the channel in which the second spindle occurs. The data shown in ***H*** are similarly represented for spindles that occur in four or more channels.

Inspection of the topography of simultaneous spindles using our visualization method shows that sheep vary in channel connectivity for both paired simultaneous spindles (Fig. 3*B*) and for four or more simultaneous spindles (Fig. 3*C*). During sleep, there were ∼50% more simultaneous spindles (Fig. 3*F*, Extended Data Fig. 4-1) than local spindles (Fig. 3*F*, Extended Data Fig. 4-1). Taking the means of the simultaneous spindle connectivity maps shows dominance of the A1 channels (Fig. 3*D*,*E*). Dominance of the A1 channels is also visible in Figure 3, *G* and *H*, where each row represents the proportions of simultaneous spindles relative to the total in each channel separately. Here, the A1 dominance can be seen such that, even in the posterior channels, relative to their own maximum, they have a high propensity to share simultaneous spindles with the A1 channels. This A1 dominance appears to be as strong as the propensity for spindles to occur simultaneously in their neighboring channels.

**Figure 4.**
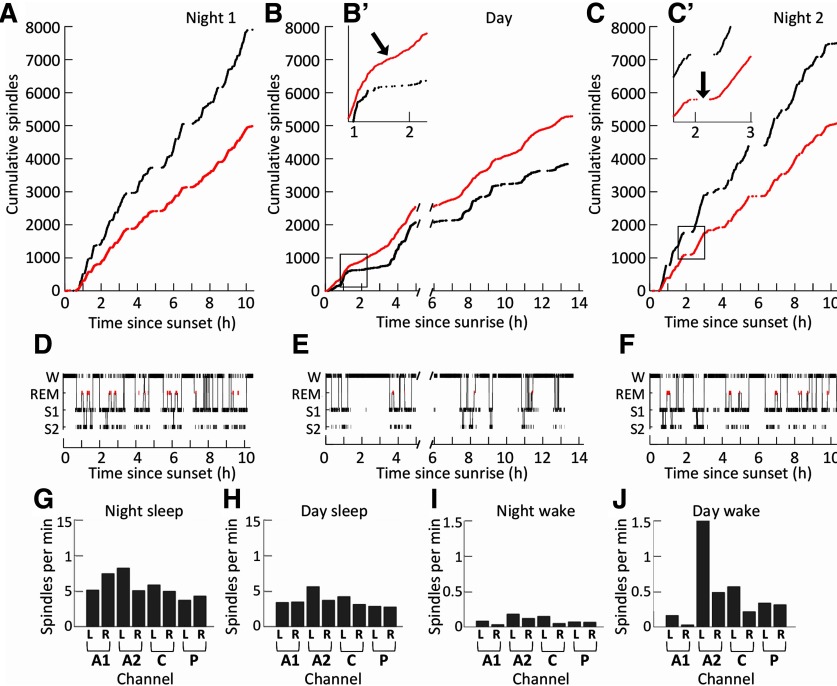
Spindles during the night and day for a single sheep. ***A–C***, Cumulative spindle plots show the rate of spindle occurrences during night 1 (***A***), day 1 (***B***), and night 2 (***C***). ***B′***, The change in local and simultaneous spindle density as day sleep returns to day wake can be seen in the enlarged plot (indicated with an arrow). ***C′***, A similar shift from sleep to wake, but in the night. Simultaneous spindles (occurring in more than one channel at a time) are shown in black. Local spindles are shown in red. ***D–F***, Hypnograms show the changes in vigilance stage for the same sheep, during night 1 (***D***), day 1 (***E***), and night 2 (***F***). ***G–J***, Spindle density in each channel for this sheep is shown for night 2 NREM sleep (***G***), day 1 NREM sleep (***H***), night 2 wake (***I***), and day 1 wake (***J***). The breaks in the data and axes at 5 h in the day recordings for ***B*** and ***E*** indicate the times at which battery changes occurred. This figure is extended in Extended Data Figures 4-1 and 4-2.

10.1523/ENEURO.0410-19.2020.f4-1 Figure 4-1 Paired Wilcoxon rank sum tests between simultaneous spindles (sim.) or local spindles, and between vigilance group for differences in spindle density (per minute). Download Figure 4-1, DOCX file.

### Simultaneous versus local spindle–SW power

Simultaneous spindles (more than one spindle occurring simultaneously) were associated with more powerful SWs than local spindles (*F*_(1,269)_ = 543, *p* < 0.0001). The mean simultaneous spindle SW peak-to-peak power was 0.085 ± 0.0031 mV, while the mean for local spindles was 0.073 ± 0.0027 mV.

### Night/day and sleep/wake differences

Spindle densities were similar for both nights (Fig. 4*A*,*C*). More spindles occurred during NREM sleep than in wake. Within NREM sleep, simultaneous spindles occurred more often than local spindles (Extended Data Fig. 4-1). Within wake, however, there were more local spindles than simultaneous spindles. Interestingly, there were far more day wake spindles than there were night wake spindles (Fig. 4*B*′,*C*′, arrows). These day wake spindles tended to occur locally and predominantly in specific channels (Fig. 4*J*), although the identity of these high-density channels was not consistent between different sheep. The channel bias in high spindle density was not apparent during NREM sleep in any of the sheep (Fig. 4*G*,*H*).

10.1523/ENEURO.0410-19.2020.f4-2 Figure 4-2 Correlations in spindle characteristics between night/day and sleep/wake periods. ***A***, ***B***, Spindle density (per minute) correlations are shown between night sleep and day sleep (***A***), and between night sleep and day wake (***B***). Spindle density is plotted separately for all eight channels in each sheep. Each sheep is identified by a unique symbol: ○, ●, □, ■, △, or ▲. Solid red lines show the linear regression. Dashed red lines show the 95% confidence bounds. ***C***, ***D***, Mean spindle frequency correlations are shown between night sleep and day sleep (***C***) and between night sleep and day wake (***D***). ***E***, ***F***, Mean spindle duration correlations are shown between night sleep and day sleep (***E***) and between night sleep and day wake (***F***). Download Figure 4-2, TIF file.

Spindles detected in wake during the day were distinct from those detected during NREM sleep in many respects (Extended Data Fig. 4-2). Day NREM sleep spindles and night-time NREM sleep spindles were highly similar in terms of spindle density (Extended Data Fig. 4-2*A*; *t* = 13.05, *p* < 0.001), frequency (Extended Data Fig. 4-2*C*; *t* = 11.64, *p* < 0.001), and duration (Extended Data Figure 4-2*E*; *t* = 3.72, *p* < 0.001). Day wake spindles and day NREM sleep spindles were similar only in spindle duration (Extended Data Fig. 4-2*F*). There was no correlation between any other characteristics of day wake spindles and day NREM sleep spindles (Extended Data Fig. 4-2*B*,*D*,*H*).

**Figure 5.**
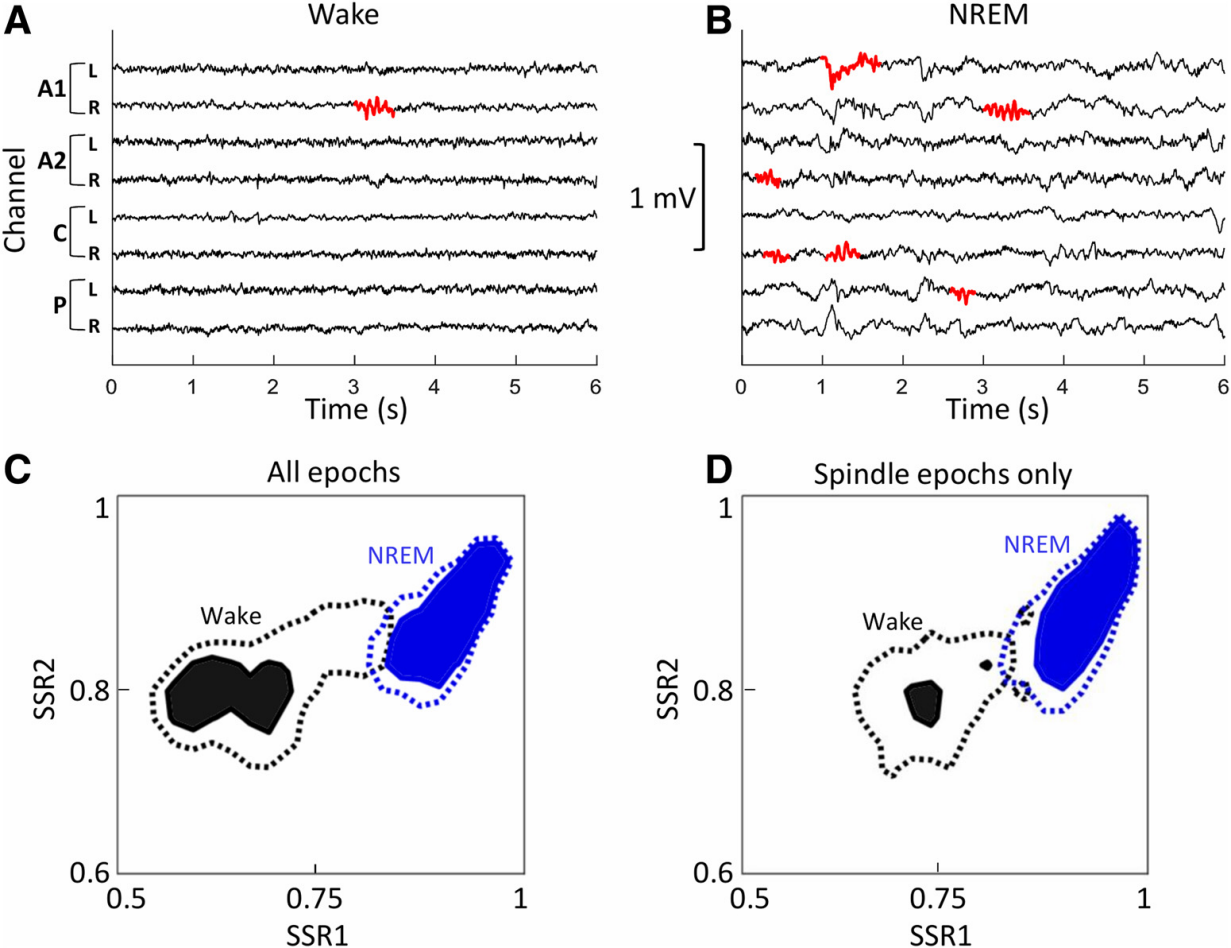
Clear separation of daytime EEG spectral states in epochs containing spindle detections. ***A***, ***B***, Local spindles were detected during the day in both wake (***A***) and NREM sleep (***B***). ***C***, Averaged sleep space state maps of spectral power during the day (all sheep and all channels) show a clear separation of wake (black) and NREM sleep (blue) states in epochs in which spindles were detected. The solid areas and dashed lines show the ratios where the two sleep states are dominant [with a threshold of 5 times (solid) or 2.5 times (dashed) above baseline]. ***D***, The separation of the solid filled areas remains clear when only epochs from individual channels in which a spindle was detected are included (all sheep, spindle channels only). SSR1 is the ratio of power of 6.5–9/0.5–9 Hz; SSR2 is the ratio of power of 0.5–20/0.5–100 Hz.

It was apparent that local spindle detections during wake could occur without any significant SW activity (Fig. 5*A*). By contrast, local spindles recorded during the day but detected during epochs scored as NREM sleep were embedded in SW activity (Fig. 5*B*). State space mapping of all scored wake and NREM sleep epochs during the day showed a very clear separation between wake and NREM sleep states with little overlap (Fig. 5*C*). When considering only epochs that contained spindles, the clear separation between the states remained (data not shown). To assess whether spindle detections during wake occurred during episodes of local sleep, the same sleep state analyses were performed using only the spindle-containing channel. Again, an obvious separation between wake and NREM sleep remained (Fig. 5*D*), further supporting our finding that spindle events can be found during clear wakefulness.

## Discussion

We have conducted a comprehensive characterization of sleep spindles in sheep. Although sleep spindles have been reported in many animal species at a wide range of different frequencies [e.g., mouse, 8–16 Hz (Kim et al., 2015); rat, 12–14 Hz (Sitnikova et al., 2012); dog, 12–15 Hz (Jeserevics et al., 2007); cat, 7–14 Hz (Paré et al., 1987); sloth, 6–7 Hz (Voirin et al., 2014); opossum, 8–11 Hz (Van Twyver and Allison, 1970); and monkey, 12–18 Hz (Takeuchi et al., 2016)], a detailed characterization of spindles in most of these species is lacking, primarily because of the challenges of recording EEG in animals. Our long-duration high-quality recording enabled a direct comparison of sheep sleep spindles with those seen in humans. Sleep spindles in sheep are similar to those found in humans in many respects, including their frequency range (10–16 Hz), duration (between 0.5 and 3 s), and density of (1–10/min) within NREM sleep (Purcell et al., 2017).

A better understanding of the role and function of sleep spindles is needed, not only in normal subjects but also in individuals with neurological conditions, particularly those in which cognitive function is abnormal. It would be extremely useful to be able to determine how characteristics of spindles change with age and whether or not they can be used as biomarkers of disease. Unfortunately, the large variation in spindle density, power, and frequency between individuals means that changes in these characteristics are difficult to measure in humans unless large cohorts are used (Purcell et al., 2017). We found that such individual differences exist in sheep. This reduces the usefulness of spindles in cross-sectional studies as a diagnostic biomarker, unless the impairments are severe. It has been suggested, however, that by taking advantage of the high intra-individual night-to-night consistency (Cox et al., 2017) it would be possible to use spindles to track progression of neurodegeneration longitudinally (Clawson et al., 2016; Seibt et al., 2016). Spindle density has been reported to change during aging (Purcell et al., 2017) as well as during neurodegenerative diseases such as Parkinson’s disease (Emser et al., 1988), Alzheimer’s disease (Kam et al., 2019), and Huntington’s disease (Wiegand et al., 1991). Indeed, there is *post hoc* evidence to suggest that sleep spindles can be used as early predictors of neurodegenerative disorders (Latreille et al., 2015). We have shown that, in sheep, despite the considerable individual variation, spindle characteristics remain consistent from night-to-night. We have developed a novel methodology for investigating the topology of simultaneous spindle occurrences. In humans, it is thought that the topology of simultaneous spindles indicates underlying information about the connectively of the brain, which is therefore highly sensitive to neurodegeneration (De Souza et al., 2016). Using our method, we saw simultaneous spindles more often in the anterior recoding channels, as is also found in humans (Botella-Soler et al., 2012). Over longer timescales, a change or breakdown in simultaneous spindle topology may indicate structural brain changes as a result of development, aging, or disease (Clawson et al., 2016). Our visualization of simultaneous spindle data can highlight such changes. Furthermore, spindles occurring in specific regions of the brain may occur because those specific brain regions of the brain are important in memory and learning tasks (Yordanova et al., 2017). Therefore, short-term increases in spindle density relative to other regions may indicate an intact ability to learn and reprocess memories. This is detectable using our simultaneous spindle visualization methodology.

A key component of spindle generation is considered to be their association with SWs. SWs are thought to synchronize simultaneous spindle events (Andrillon et al., 2011), which underlie mechanisms of memory consolidation during sleep (Kim et al., 2017; Yordanova et al., 2017). In accord with findings in both humans (Andrillon et al., 2011) and mice (Kim et al., 2015), our results showed that simultaneous spindle events were associated with stronger SWs than local spindle events. Interestingly, during wake in the day we found a surprising number of these local spindle events. Spindles are generally considered to be events that occur only during sleep. We exclude the possibility that these wake spindles were occurring during transient periods of sleepiness or during local sleep because epochs in which they occurred were undeniably wake-like. These wake spindles also appeared to occur predominantly in single channels, although there was no consistency between sheep in which channel they would dominate. Furthermore, the characteristics of wake spindles differed from those detected during sleep. While it is possible that wake spindles are a sheep-specific phenomenon, we think this is unlikely given the similarity of sheep sleep spindles to those detected in humans and other animals. Rather, given the lack of previous descriptions of spindles during wake, it is more likely that oscillations in this frequency range during wake are usually classified as alpha activity. Some other studies show that characteristics of alpha activity during wake are different from those of sleep spindles (Barman et al., 1995; Başar et al., 1997). Alpha activity is historically considered to be associated with the idling state of the brain in wake, but there are a growing number of studies linking alpha waves to many elements of sensory and cognitive processing (Palva and Palva, 2007; Başar, 2012; Sadaghiani and Kleinschmidt, 2016). These processes include memory retrieval, attention, and consciousness (Klimesch, 2012; Kwok et al., 2019), and are related to behaviors that have been well studied in animal models (Bizon et al., 2012; Boly et al., 2013; McBride and Morton, 2018). Given that both alpha oscillations and sleep spindles are associated with memory and cognitive processing, the possibility that the wake spindles we detect play a role in daytime cognitive processes is intriguing. In humans, rare reports of sleep spindles during wake are considered to be a sign of abnormal brain function or aging (Iyama et al., 1992). It would be particularly interesting to investigate the presence of wake spindles in neurodegenerative diseases involving the caudate nucleus and cortex, such as Huntington’s disease, where an increase in sleep spindles has been reported (Wiegand et al., 1991).

To date, long-term EEG monitoring in human subjects in order to track neurodegeneration has not been validated. Sheep would make an excellent species for tackling such challenges, particularly since long-term (up to 4 years) stable recordings can be made in sheep, and they are also an established model for studying human neurologic disease and dysfunction (Perentos et al., 2016; Morton, 2018). This work provides baseline details of sleep spindle characteristics in sheep, as well as methodology that can be used to investigate changes in these characteristics over time.

## Synthesis

Reviewing Editor: Bradley Postle, University of Wisconsin Decisions are customarily a result of the Reviewing Editor and the peer reviewers coming together and discussing their recommendations until a consensus is reached. When revisions are invited, a fact-based synthesis statement explaining their decision and outlining what is needed to prepare a revision will be listed below. The following reviewer(s) agreed to reveal their identity: NONE.

Here is the full text of the two reviews:

R1:

I think this is a careful and detailed description of sleep spindles in sheep that lays the groundwork for establishing this species as a model for investigating the functions of spindles, and their relationship to neurodegenerative diseases in humans. The paper is well written and presented. The data has been carefully obtained and analyzed.

The following comments should be addressed:

The authors note that sheep can sleep while ruminating, as described in previous studies. They also mention that the characteristics of spindles did not differ between NREM sleep with and without rumination. Was the EEG entirely free from fast EMG and slow genioglossal artifacts during rumination? If not, how were the artifacts handled? Along these lines, it would be useful to provide a figure showing raw recordings for all states.

The authors mention that the electrooculogram (EOG) was recorded “from the eye muscles.” The EOG actually detects field potentials resulting from the movement of the eye ball, due to the retina being more negative than the cornea. While it is true that these movements obviously depend on muscle activity, the muscles are not being recorded directly with the EOG.

Page 7, second full sentence: Change “...that was then subtracting this from each EEG channel.”, to “...that was then subtracted from each channel.”

The voltage scale for Fig 7 and the amplitudes of slow waves mentioned in the text (peak-to-peak 0.085 uV) seem too small. I would expect much higher voltages from these recordings. Did you mean mV instead? Also, Fig 1D and E need a voltage scale.

The electrodes site names (F, C, and O) and their general position relate to the nomenclature used in human EEGs. However, it is not clear to me that all frontal “F” electrodes in sheep are recording from cortical areas homologous to those that frontal (F) electrodes record in humans. If the authors can not provide strong evidence showing that they are in fact recording from homologous regions, then I would recommend a different nomenclature to avoid the implication that they are homologous. For example, you could use anterior, aterior-medial, posterior- medial, and posterior, or something along those lines.

The authors report that spindles were usually associated with the down-state of cortical slow oscillation derived from the EEG signal. This is opposite from what has been found in humans. This finding is also surprising because the cortical network should be largely quiet during the hyperpolarized down-state of the slow oscillation in membrane potentials, and therefore incapable of generating oscillations such as spindles. Are you sure that your interpretation of the relationship between EEG slow waves and the state of the underlying cortical membrane potentials is correct?

R2

The paper presents new data on sleep spindles in sheep. The authors made unique experiments with wireless EEG recordings during two nights and one day in six sheep. Results are presented in excessive details which, in some parts of the paper, make it difficult to read. Although the presence of too many details, some procedures are still not clear how they were exactly performed.

Questions

(1) Spindle detection.

- Although the algorithm for spindle detection is described in detail, it is interesting to know if these criteria were arbitrary or taken from any recommended literature source. Please indicate.

- How was exactly FFT performed? Did you use spectral power density, relative power or what? As I understand, FFT was applied to the detected spindles only. How did you deal with the different frequency resolution? For example, if the spindle duration was 0.3 s, then the frequency resolution was 1/0.3=3.33 Hz, i.e. the whole spindle frequency range (10-16 Hz) was described by less than 2 points. This prevents from group averaging and obtaining more reliable results.

- Please provide argumentation for dividing spindle grouping into three bands (5-10, 10-13, and 13-16 Hz).

- The ANOVA results about individual variance do not indicate if a single subject was deviant or the variance was overall high. This information is important to support the claim that individual variance was high.

(2) Sleep stages.

Please provide a reference to verify the validity of wake/sleep scoring in sheep. The way the study of Perentos et al. (2016) is referred does not inform if this is the source about criteria for the identification of 7 wake/sleep stages in sheep and for the algorithm for semi-automatic sleep stage scoring.

(3) Spindle-Slow wave phase angle coupling.

- Likewise, please explain how exactly the following approach “We determined spindles to be associated with a SW if the nearest SW peak-to-peak amplitude to the spindle centre was more than 150% of the mean SW peak to peak amplitude.” was chosen for evaluation of spindle-SW association and in which reference this approach was validated?

- Initially, in the text, you explained about SW peak-to-peak amplitude measures, then the description changes for SW power. Please specify.

- Which value of phase angle was accepted as evidence for spindle-SW coupling?

(4) Simultaneous spindles.

How was it dealt with the case when different pairs of channels showed presence of spindles? For further evaluation, everything was pooled together or?

(5) State-space analysis.

How did you define the band ranges for SSR1 and 2? In the paper of Diniz Behs et al., 2010, it is clearly stated that the choice of band ranges was empirically defined with the expectation that 6.5-9 Hz is the dominant EEG frequency in mice. Why did you use the same frequency range? Did you expect domination of theta frequencies in the sheep EEG? How many epochs were included in the analysis for ‘all epoch’ state map?

(6) Statistical analysis.

- Did you use repeated measures ANOVA? Which level was accepted for significance? In the results, original or corrected degrees of freedom were reported? How were power values used for statistical analysis - did you perform any normalization procedures?

- I am not sure if it is correct to treat separate individuals as levels of a variable in ANOVA.

(7) Did you compare topography between slow and fast spindles?

(8) Legends and Figures.

Fig. 1. Proportion of detection threshold is not clear.

Fig. 2. Please add additional indication for NREM sleep in C (e.g., S1 - U2 in a circle).

Fig. 3. Where are the channels in A?

Fig. 4. What is the information behind Fig. 4? Do the colored plots in G and H present symmetric matrices? How can you explain that, e.g. F1 - O horizontally is different from O - F1 vertically?

Fig. 5. How can it be explained that the number of spindles during the day wake is dramatically increased at F2-Left?

Fig. 6. To my opinion this figure is redundant and can be skipped.

Fig. 7. What is the interpretation of plots in C and D? It is tоo complicate to understand the meaning of the position of the NREM spot.

Major

(1) The objectives of the study to characterize sleep spindles in sheep are very generic. The authors have to provide more arguments why this characterization is relevant. The idea to create a useful animal model to provide comparisons with human studies is not clearly formulated, although the discussion contains various implications in this direction.

(2) There is tоo much information included in the paper. I understand that experiments with sheep are very seldom, but please filter the whole information and include in the main text only valuable results. All other measures can be included in Supplementary Material. Because of the multitude of investigated parameters (spindle density, coupling, locality, etc.) and the multitude of factors tested (individual, day/night, stage, macrostructure, etc.), to achieve clarity I would suggest presenting parameters, factors and related schematic results in a structured manner (e.g., in a table). The current presentation of various parameters/factors is not comprehensive. Also, the authors may decide to re-structure results presentation by defining physiological rather than technical/statistical clusters of results exposition.

(3) The Discussion in its present form is highly speculative and needs to be re-structured to identify clear foci related to spindle characteristics. Also, references to human cognition are confusing. Please rework the Discussion section by shortening and making it concise and strictly related to the Results.

(4) In the paper, you demonstrated lots of differences between sheep and humans, so that I would propose to avoid conclusions like usefulness of sheep investigations instead of human studies. Please use with caution terms like cognitive processing in animals.
