# Supplementary figures and images for: Characterizing Sleep Spindles in Sheep
Source: eNeuro. 2020 Mar 6;7(2):ENEURO.0410-19.2020. doi: 10.1523/ENEURO.0410-19.2020 (PMC7082130; doi:10.1523/ENEURO.0410-19.2020)

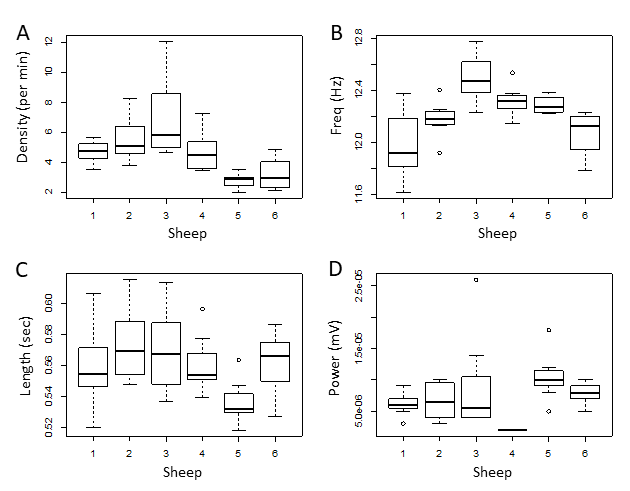

Supplement: Figure 1-1 — Examples of intersheep differences in spindle characteristics. Whisker plots show spindle characteristics during NREM for each sheep (N = 6). A–D, Spindle density (A) frequency (B), spindle length (C), and spindle power (D). All data are taken from night 2. Spindle characteristics vary widely between individuals, and this is not due to a single outlier. Download Figure 1-1, TIF file. [file enu-eN-NWR-0410-19-s02.tif]

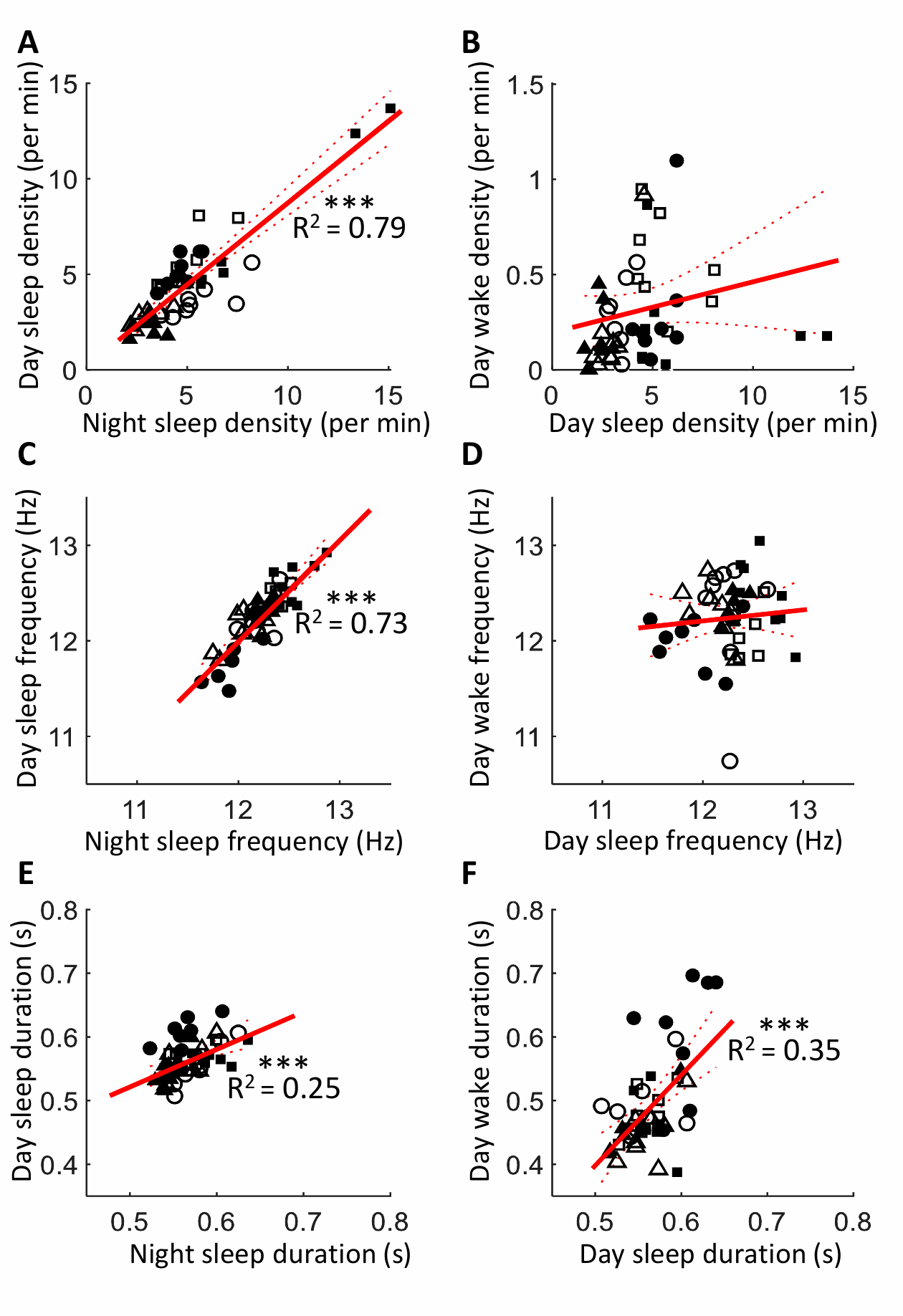

Supplement: Figure 4-2 — Correlations in spindle characteristics between night/day and sleep/wake periods. A, B, Spindle density (per minute) correlations are shown between night sleep and day sleep (A), and between night sleep and day wake (B). Spindle density is plotted separately for all eight channels in each sheep. Each sheep is identified by a unique symbol: ○, ●, □, ■, △, or ▲. Solid red lines show the linear regression. Dashed red lines show the 95% confidence bounds. C, D, Mean spindle frequency correlations are shown between night sleep and day sleep (C) and between night sleep and day wake (D). E, F, Mean spindle duration correlations are shown between night sleep and day sleep (E) and between night sleep and day wake (F). Download Figure 4-2, TIF file. [file enu-eN-NWR-0410-19-s05.tif]
