# Supplementary material for: Characterizing Sleep Spindles in Sheep
Source: eNeuro. 2020 Mar 6;7(2):ENEURO.0410-19.2020. doi: 10.1523/ENEURO.0410-19.2020 (PMC7082130; doi:10.1523/ENEURO.0410-19.2020)
Supplement: Figure 1-2 — General spindle characteristics in sheep. Download Figure 1-2, DOCX file. [file enu-eN-NWR-0410-19-s03.docx]

**Extended Data 3**

**Figure 1-2. General spindle characteristics in sheep.**

| **Spindle Characteristic** | **Mean values for each channel (+- s.e.m)** | | | | | | | |
| --- | --- | --- | --- | --- | --- | --- | --- | --- |
|  | **A1-L** | **A1-R** | **A2-L** | **A2-R** | **C-L** | **C-R** | **P-L** | **P-R** |
| **Density (per min of NREM)** | 5.66 (1.31) | 5.90 (1.30) | 4.84 (0.95) | 4.65 (0.33) | 4.47 (0.77) | 4.30 (0.38) | 3.50 (0.34) | 3.90 (0.31) |
| **Frequency (Hz)** | 12.32 (0.10) | 12.30 (0.08) | 12.34 (0.05) | 12.14 (0.07) | 12.19 (0.10) | 12.06 (0.13) | 12.18 (0.08) | 12.22 (0.10) |
| **Power (mV x 10^5^)** | 1.07 (0.38) | 0.90 (0.18) | 0.60 (0.13) | 0.62 (0.15) | 0.47 (0.08) | 0.63 (0.13) | 0.62 (0.10) | 0.68 (0.13) |
| **Length (s)** | 0.57 (0.01) | 0.58 (0.01) | 0.55 (0.01) | 0.56 (0.01) | 0.55 (0.01) | 0.56 (0.01) | 0.57 (0.01) | 0.56 (0.01) |

s.e.m = Standard error of the mean. A = anterior, C = central, P = posterior, L = left, R = right.
