# Supplementary material for: Characterizing Sleep Spindles in Sheep
Source: eNeuro. 2020 Mar 6;7(2):ENEURO.0410-19.2020. doi: 10.1523/ENEURO.0410-19.2020 (PMC7082130; doi:10.1523/ENEURO.0410-19.2020)
Supplement: Figure 4-1 — Paired Wilcoxon rank sum tests between simultaneous spindles (sim.) or local spindles, and between vigilance group for differences in spindle density (per minute). Download Figure 4-1, DOCX file. [file enu-eN-NWR-0410-19-s04.docx]

**Extended Data 4**

**Figure 4-1. Paired Wilcoxon rank sum tests between simultaneous or local spindles, and between vigilance group for differences in spindle density (per minute).**

| **Spindle group** | **Density**  **(spindles per min)** | **s.e.m** | **Spindle group** | **Density**  **(spindles per min)** | **s.e.m** | **p value** | **Test stat (W)** |
| --- | --- | --- | --- | --- | --- | --- | --- |
| **Night/night comparisons** |  |  |  |  |  |  |  |
| night sleep sim. | 3.0376 | 0.2851 | night wake sim. | 0.0224 | 0.0044 | < 0.001 | 2304 |
| night sleep local | 1.8491 | 0.0921 | night wake local | 0.0661 | 0.0091 | < 0.001 | 2304 |
| night sleep sim. | 3.0376 | 0.2851 | night sleep local | 1.8491 | 0.0921 | < 0.001 | 1710 |
| night wake sim. | 0.0224 | 0.0044 | night wake local | 0.0661 | 0.0091 | < 0.001 | 508 |
| **Day/day comparisons** |  |  |  |  |  |  |  |
| day sleep sim. | 2.5845 | 0.2543 | day wake sim. | 0.0580 | 0.0070 | < 0.001 | 2304 |
| day sleep local | 1.8460 | 0.1114 | day wake local | 0.2600 | 0.0427 | < 0.001 | 2286 |
| day sleep sim. | 2.5845 | 0.2543 | day sleep local | 1.8460 | 0.1114 | 0.04705 | 1423 |
| day wake sim. | 0.0580 | 0.0070 | day wake local | 0.2600 | 0.0427 | < 0.001 | 485 |
| **Night/day comparisons** |  |  |  |  |  |  |  |
| night wake local | 0.0661 | 0.0091 | day wake local | 0.2600 | 0.0427 | < 0.001 | 513 |
| night wake sim. | 0.0224 | 0.0044 | day wake sim. | 0.0580 | 0.0070 | < 0.001 | 615 |

Sim. = simultaneous, W = Wilcoxon rank test statistic, s.e.m = Standard error of the mean.
